# Laryngology

**Published:** 1895-12

**Authors:** Barclay J. Baron


### LARYNGOLOGY.

The surgical treatment of laryngeal tuberculosis formed the subject of a discussion at the British Laryngological and Otological Association at its meeting in July, 1895.[1]

Krause's remarks may be thus summed up: (1) *The choice of cases.* He does not advise extreme carefulness. Ease can be given even in the most advanced or desperate cases, and neither high fever nor great debility need prevent our giving the patient this relief. He quotes a very bad case in which the excision of large pieces from the ary-epiglottic and pharyngo-epiglottic folds was followed by remarkable benefit, owing to swallowing becoming practically painless. (2) *The performance of the operation.*—The parts most diseased, and the disease of which most endangers nutrition or respiration, should be attacked first. Gradually the whole of the diseased structures can be cut away, not excepting the epiglottis or the arytenoid cartilages. Deep and thorough operation is necessary, and after it lactic acid, well rubbed into the wounded surface, is the best substance that can be used. Sulphoricinate of phenol, as introduced by Ruault, has a similar but slower effect than lactic acid. (3) *Relapses.*—When strength is well preserved and tendency to cicatrisation is observed we get lasting results as often as in the lungs. Krause has seen cases in which the larynx has remained well, although the disease in the lungs has continuously progressed. In fact, as a rule, after the introduction of the surgical treatment laryngeal relapses are rarer than pulmonary ones.

Heryng's remarks may be summarised as follows: (1) Laryngeal phthisis can heal by itself without any local treatment. The ulcers on the vocal cords and the posterior wall of the larynx heal most frequently; very rarely those more serious cases, in which the infiltration and proliferation products are attended with deep ulceration, as also those cases in which the disease has spread to the cartilage and has led to deep disintegration. (2) The objects of the treatment are: First and most important, the relief of the dysphagia; secondly, the relief of stenosis; thirdly, the cure of aphonia. (3) *Surgical treatment is indicated:* (*a*) In tubercular tumours of the epiglottis. (*b*) In circumscribed chronic tumour-like infiltrations of the posterior wall of the larynx, which show little inclination to break down. (*c*) In chronic tumours resting on an inflammatory base, surrounded with proliferation products, which resist all other methods of treatment. (*d*) In partial disease of the larynx, when epiglottis, false cords, and lateral ligaments are affected. (4) *Surgical treatment is contra-indicated:* (*a*) In advanced phthisis of the lungs, with hectic and wasting. (*b*) In diffuse miliary tubercle of the larynx or, rather, of the larynx and pharynx. (*c*) In all cachetic conditions. (*d*) In severe stenosis of the

[1] *J. Laryngol.*, 1895, ix. 572.

larynx, caused by inflammatory swelling of the affected parts. In these cases tracheotomy must be performed as soon as possible. (*e*) In patients exhibiting fear and nervous excitability, mistrust of the physician, and who are always changing their doctor, especially those whose condition promises little hope of recovery. (5) With the proper application of cocaine, the operation itself is not painful. Submucous injection of cocaine is hardly ever necessary. (6) Pyoktanin (one to two per cent. solution) has proved a very good means of preventing inflammation in the parts operated on. It must be applied to the surface of the wound twice a day. (7) Recurrence takes place frequently at the place operated on, sometimes at a little distance from it. It is explained not only by inaccessibility of certain parts of the larynx to our instruments, but also by the imperfect performance of the operation. In most cases, however, the recurrence is due to the disease spreading to the lungs, and the insufficient power of resistance to the infection. (8) Nearly the whole of the upper part of the larynx is accessible to surgical treatment by suitable instruments. It should be a rule in surgical treatment to excise as much of the affected parts as possible at one sitting. The double curette has the advantage over the single curette in certain cases. (9) It must be explained to the patients and their friends, before the operation, that the dysphagia cannot be removed at once by surgical interference; that it is very often increased for a few days, and, further, that the operation does not effect a radical cure. It is also advisable to tell the patients that the radical removal of the accessible parts is very seldom successful in one sitting; that, in spite of a successful operation, the disease of the larynx may return, and that the physician can give no guarantee of ultimate restoration to health. (10) The conditions of success of the surgical treatment in general depend: (*a*) On the local character of the affection, its extent and its nature. (*b*) On the general state of the patient, his nutrition and his strength. (*c*) On the anatomical character of the affection of the lungs. (*d*) On the age of the patient, his constitution, his profession, his material circumstances, his temperament, and his character. (*e*) On the thoroughness of the operation itself and the skill of the operator, as well as on the localisation of the processes in such places where it is technically possible to perform a radical extirpation of the infected tissues. (*f*) On the careful after-treatment, and the capability of the patient to submit to a prolonged dietetic and climatic treatment.

Gerhardt [1] says we can obtain important information by external examination of the larynx and trachea. During dyspnœa, if the larynx move only slightly, the cause is to be sought in the trachea; if more extensively, the stenosis is in

[1] *J. Laryngol.*, 1895, ix. 329.

the larynx. In laryngeal croup the neck is bent backwards so that the bodies of the vertebræ may press the cartilages of the larynx flat; in stenosis of the trachea the chin approaches the breast. In tracheal stenosis there is expiratory stridor; in laryngeal stenosis, inspiratory stridor. The author does not look upon tracheal tugging as a characteristic symptom of aneurysm. In a case of aneurysm of the ascending aorta he observed distinct interruption of the tone during prolonged phonation. If a finger be placed on each side of the larynx, between the thyroid and cricoid cartilage, the action of the crico-thyroid is felt. If the contraction be absent on one side, a paresis is present, and we can determine whether this is of central or peripheral origin by electrical examination. If the finger be pushed higher, towards the thyroid cartilage, the vibrations of the vocal cords are felt; in this way a paralysis of the recurrent can be diagnosed in many cases, and the presence of extensive ulcers and tumours be established. The voice can sometimes be improved by pressing on the thyroid cartilage, especially by compression in hysterical paresis. If "ah" or "oh" can be produced by pressing on the thorax—passive voice production—then we have probably to deal with a double abductor paralysis. Abnormal movements of one vocal cord can also be felt with the finger placed behind the upper cornu of the thyroid. Gerhardt refers to a case of sarcoma of the leg, and of the right and left lung and pleura, in which there was giddiness on standing erect. With the laryngoscope, tremor-like adduction movements of the vocal cords were seen during expiration, which could also be felt externally. The *post-mortem* examination revealed a sarcoma metastasis in the frontal bone which pressed on the frontal convolutions.

At the Laryngological Society of London on October 10th, 1894, Lake [1] showed two cases of lupus of the nose and palate treated by thyroid extract. One was a boy, 11 years of age, who had suffered for fourteen months, and whose soft palate and posterior pillars were also affected. He had taken $7\frac{1}{2}$ grains of thyroid extract daily for three months, and was very much improved. The second case was a girl, 16 years old, who had been affected for three years. She took $17\frac{1}{2}$ grains daily for the same period of time, and was very much improved.

Spencer Watson [2] relates particulars of a case in which tachycardia was associated with, and apparently largely dependent on, nasal polypi. Removal of the growths removed all cardiac trouble, which had not returned within three months of the operation.

I have treated two cases of epilepsy, in one of which there were associated nasal polypi, the removal of which greatly

[1] *J. Laryngol.*, 1895, ix. 165. [2] *Brit. M. J.*, 1895, ii. 1097.

ameliorated the condition; in fact, practically no fits occurred until recurrence of the growth, further operation on which again stopped them. In the other case, snaring off pieces of the greatly hypertrophied inferior turbinated bodies, and the application of the galvano-cautery, completely stopped the fits for eight months.

It is, therefore, of importance to examine the nose for possible distribution of reflex disturbance.

Milligan [1] draws attention to the question of vocal defects amongst school board teachers, with special reference to the occurrence of teachers' nodes. All who are examining the larynx in teachers and other voice users will agree that this is a most important matter. Milligan rightly insists on the necessity of raising the age when girls begin the severe vocal duties of pupil teachers. Better ventilation of class-rooms; having the benches placed in tiers one above the other, so that the teacher speaks with head erect; and, above all things, efficient teaching of voice-production before the girls commence their work are all prophylactics. When the larynx is congested and nodes are present, physiological rest, the local application of weak astringents, and the nightly application of Leiter's cold coil for an hour at a time, are all valuable. If the nodes are large enough the galvano-cautery, or even the forceps, may be used to remove them.

In this department of work great mischief is being done throughout the country. The female teachers and, to a less extent, the males are, as I know from my own practice, in many cases incapacitated from following their occupation. The main causes of the serious laryngeal mischief that develops are faulty methods of voice-production, too large classes, and ill-ventilated rooms. Prevention ought not to be difficult; cure is, in many cases, nearly or quite impossible.

BARCLAY J. BARON.

### OTOLOGY.

[1] *Brit. M. J.*, 1895, ii. 1097.

